# Sirtuin 6 Protects Against Oxidative Stress and Vascular Dysfunction in Mice

**DOI:** 10.3389/fphys.2021.753501

**Published:** 2021-10-20

**Authors:** Lawrence E. Greiten, Bin Zhang, Carolyn M. Roos, Michael Hagler, Fritz-Patrick Jahns, Jordan D. Miller

**Affiliations:** ^1^Department of Surgery, Mayo Clinic, Rochester, MN, United States; ^2^Department of Biomedical Engineering and Physiology, Mayo Clinic, Rochester, MN, United States

**Keywords:** sirtuin 6, histone deacetylation, vascular function, endothelial dysfunction, aging

## Abstract

**Objective:** Sirtuin deacetylases are major regulators of organismal aging, and while depletion of sirtuin 6 (SIRT6) in mice results in a profound progeroid phenotype, the role of SIRT6 in the regulation of vasomotor function is unknown. Thus, our objective was to test the hypothesis that reductions in SIRT6 elicit endothelial dysfunction in young, genetically altered mice.

**Results and Approach:** We used young (3 month old), littermate-matched, SIRT6 wild-type (WT), and SIRT6 heterozygous (HET) mice. SIRT6 expression (qRT-PCR) was reduced by 50% in HET mice. Carotid vessel responses to acetylcholine, sodium nitroprusside, U46619, and serotonin were examined in isolated organ chamber baths. Relaxation in response to acetylcholine (ACH) was impaired in HET mice compared to littermate-matched WT controls (67 ± 3% versus 76 ± 3%, respectively; *p* < 0.05), while responses to sodium nitroprusside were unchanged. Short-term incubation of carotid rings with the NAD(P)H oxidase inhibitor, apocynin, significantly improved in vessels from HET mice but not their WT littermates. Peak tension generated in response to either U46619 or serotonin was significantly blunted in HET mice compared to their WT littermates.

**Conclusion:** These data suggest that SIRT6 is a key regulator of vasomotor function in conduit vessels. More specifically, we propose that SIRT6 serves as a tonic suppressor of NAD(P)H oxidase expression and activation, as inhibition of NAD(P)H oxidase improved endothelial function in SIRT6 haploinsufficient mice. Collectively, SIRT6 activation and/or histone acetyltransferase inhibition may be useful therapeutic approaches to reduce endothelial dysfunction and combat age-associated cardiovascular disease.

## Introduction

A growing body of data suggests that the sirtuin deacetylases (SIRTs) play a major role in protecting against a number of age-related diseases (Herskovits and Guarente, 2013; Hubbard and Sinclair, 2014; Matsushima and Sadoshima, 2015; Chen et al., 2020). With regards to the vasculature, previous reports suggest that reduction of SIRT1 leads to increases in histone acetylation, increases in inflammation, and endothelial dysfunction due in part to nitric oxide synthase hyperacetylation and its subsequent inactivation (Kitada et al., 2016; Zhang et al., 2017). The effects of reducing other SIRT isoforms in the regulation of histone acetylation and vascular function, however, remain poorly understood.

Total genetic knock-down of SIRT6 induces age-associated phenotypes (e.g., kypholordosis and osteoporosis, etc.) and highly susceptible to DNA damage and increases in histone acetylation (Miller et al., 2008). Recent publications have indicated SIRT6 as a potential therapeutic candidate in diabetic wound healing (Kuang et al., 2018), and atherosclerotic model (Grootaert et al., 2021). For instance, Liu et al. (2016) has reported that acute knock-down of SIRT6 via lentivirus in ApoE^–/–^ high-fat diet mice caused impaired endothelial function, enhanced plaque formation, and decreased plaque stability. To our knowledge, the effects of global-genetic reductions of SIRT6 on vasomotor function remain largely unknown. Previous work has demonstrated that age-associated reductions in endothelial function are mediated in part by increases in vascular inflammation (Csiszar et al., 2008; Donato et al., 2008, 2009; Ungvari et al., 2011) and concomitant increases in NAD(P)H oxidase-derived reactive oxygen species (ROS) (Li and Shah, 2004; Brewer et al., 2006; Dworakowski et al., 2008a; Murdoch et al., 2011). While increases in NAD(P)H oxidase-derived ROS have been shown to play a causal role in induction of age-related vascular fibrosis and stiffening (Bell et al., 2005; Cave et al., 2005; Akki et al., 2009), epigenetic events regulating these pro-inflammatory cascades remain poorly understood.

Thus, our working hypothesis in the current study was that reductions in SIRT6 are an independent contributor to increases in histone acetylation and oxidative stress in blood vessels, which could place SIRT6 at the forefront of contributors to age-associated cardiovascular disease. Our specific aims were to determine: (1) whether SIRT6 regulates histone acetylation in arteries, and (2) whether endothelial dysfunction in SIRT6-deficient mice is mediated by increases in NAD(P)H oxidase-derived reactive oxygen species.

## Methods

### Animals

For these studies, we studied mice at 3 months of age (i.e., a relatively young age). For establishment of an in-house colony, mice that were heterozygous for SIRT6 (SIRT6^+/–^) mice were acquired from Jackson Laboratories (stock number: 006050) along with background strain-matched wild-type mice (SIRT6^+/+^), with SIRT6 being inactivated with a targeting vector containing lacZ and neomycin resistance genes disrupting exons 1–6. SIRT6^+/–^ mice were then bred with SIRT6^+/+^ to generate all experimental mice used for this study, additionally littermate-matched pairs were used whenever possible. Mice had free access to water and regular chow diet, and maintained on a 12-h light, dark cycle. All experimental protocols were approved by the Mayo Clinic Institutional Animal Care and Use Committee (IACUC) and conformed to guidelines set forth by the National Institutes of Health and the Guide for the Care and Use of Laboratory Animals. Animals were euthanized using intraperitoneal injection of pentobarbital sodium (>100 mg/kg).

### Vasomotor Function

Both right and left carotid arteries were carefully removed and cleaned of adventitia, then each carotid was cut into two segments, and threaded onto two stainless steel hooks. In brief, hooks were then suspended in an isolated organ bath chamber which contained warm-oxygenated (95%O_2_, 5%CO_2_) Krebs solution. Vessels were equilibrated to a baseline tension of 0.25 grams. Once stabilized, responses to acetylcholine (to assess endothelium-dependent relaxation), sodium nitroprusside (to assess endothelium-independent relaxation and sensitivity to nitric oxide donors), U46619 (a thromboxane receptor agonist to allow assessment of vascular smooth muscle cell contractile function), and serotonin (to assess vascular smooth muscle cell contractile function) were evaluated in all mice, as described previously (Roos et al., 2013). Additionally, responses to acetylcholine were also assessed in the presence of apocynin (100 μM), an inhibitor of NADPH oxidase, or incubated with L-NAME (100 μM), a nitric oxide synthase inhibitor.

### RNA Extraction and cDNA Synthesis

Thoracic aortic arch tissue was carefully taken from test animal with perivascular adipose tissue removed, snap freezing, and stored at −80°C prior to RNA extraction. RNA samples were isolated using spin columns and chloroform extractions (Ambion), as described previously (Hagler et al., 2013).

### Quantitative Real-Time RT-PCR

Quantitative real-time PCR was performed on a StepOne Plus RT-PCR machine (Applied Biosystems). Gene expression for SIRT6 and other SIRT isoforms were measured using TaqMan Gene Expression Assay primers (Life Technologies). Gene expression levels were normalized by GADPH and hypoxanthine phosphoribosyltransferase 1 (HPRT1), both commonly used housekeeper genes and expressed using the ^ΔΔ^Ct method.

### Measurement of Reactive Oxygen Species in Aorta

Levels of ROS in descending thoracic aorta were measured using lucigenin-enhanced chemiluminescence, as described previously (Miller et al., 2008). In brief, excess adventitial tissue and perivascular fat were removed, and cut into four sections of ∼4 mm in length. Cuvettes containing warm Leibovitz’s L15 medium (a CO^2–^ independent medium) and 5 μM lucigenin (N, N′-Dimethyl-9, 9′-biacridinium dinitrate, Sigma) were placed in a FB12 luminometer (Berthold) and chemiluminescence was measured for 10 min to obtain baseline measurement. Once baseline measurement was taken, the cleaned 4-sections of aorta was carefully added to the cuvette and replace back into luminometer to detect ROS levels of the vessel. Upon measurement completion of vessel alone, 100μM of apocynin was added to the vessel to observe changes in ROS due to NADPH oxidase inhibition.

### Immunohistochemistry

Descending thoracic aorta (1–2 mm length) was embedded in tissue freezing medium (Triangle Biomedical Sciences). Cross-sections of the frozen embedded tissue were subsequently cryosectioned (10μm thickness), and used for histological and immunohistochemical analyses. Slides were fixed in 2% paraformaldehyde, rinsed with TBS, permeabilized with TBS containing 0.1% Triton, and blocked with donkey serum for 1 h at room temperature. Primary antibody (H3K9/14ac, Diagenode, 1:200) was incubated overnight at 4°C and secondary antibody (Invitrogen, Alexa Fluor 647, 1:500) was incubated for 1 h (room temperature). Sections were mounted in ProLong Gold DAPI solution, and then imaged on a Zeiss 780 LSM confocal microscope at 20× magnification. Subsequently, images were quantified and analyzed using the software ImageJ (NIH).

### Statistical Analyses

Between-group differences were detected using unpaired, Bonferroni-corrected t-tests in GraphPad Prism Software. All data are expressed as mean ± SE.

## Results

### Sirtuin 6 Isoform Expression (Quantitative Real-Time RT-PCR)

**FIGURE 1:**
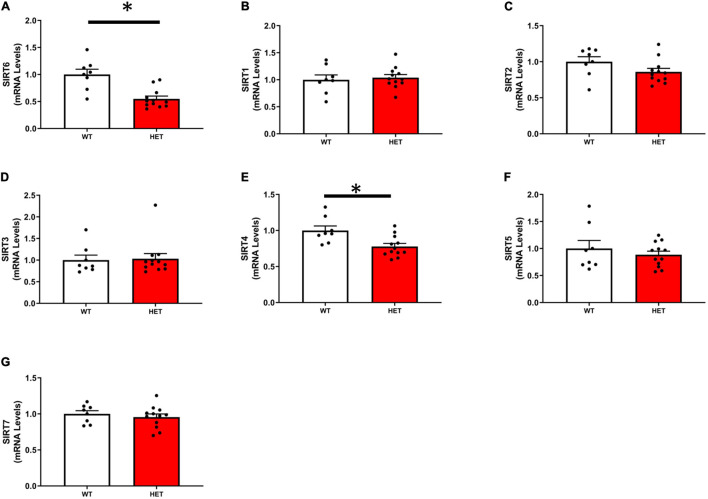
Expression of individual sirtuin isoforms in SIRT6^+/+^ and SIRT6^+/–^ mice. **(A)** Demonstrates successful significant reduction of SIRT6 expression in SIRT6^+/–^ mice by ∼50%. Expression of other sirtuin isoforms was reduced [only SIRT2 and SIRT4, **(C,E)**] or unchanged by genetic inactivation of one copy of SIRT6 **(B,D,F,G)**. (SIRT6^+/+^ *n* = 9 and SIRT6^+/–^ *n* = 12). * denotes *p* < 0.05.

As expected, the expression of SIRT6 was reduced by ∼50% in SIRT6^+/–^ mice compared to SIRT6^+/+^ mice. Of the remaining SIRT isoforms, SIRT2 and SIRT4 were decreased in SIRT6^+/–^ mice, however, only SIRT4 was significantly reduced. The expression of remaining SIRT isoforms was unchanged (Figures 1A–G).

### Histone Acetylation Levels (Immunohistochemistry)

**FIGURE 2:**
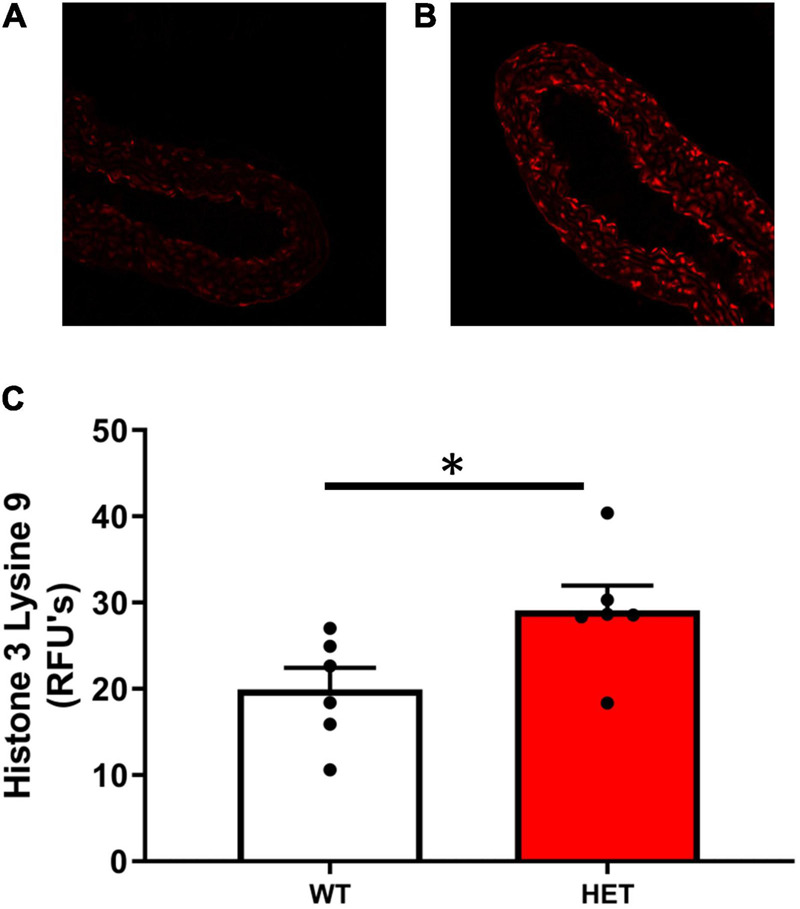
Histone acetylation levels in SIRT6^+/+^ and SIRT6^+/–^ mice. **(A–C)** Acetylation levels of histone 3 lysine 9 (H3K9) was significantly increased in aorta from SIRT6^+/–^ mice compared to SIRT6^+/+^ mice (*p < 0.05). Qualitative examination of micrographs suggested that increases in histone acetylation occurred throughout the vessel wall (i.e., in both endothelium and vascular smooth muscle layers) (SIRT6^+/+^ *n* = 6 and SIRT6^+/–^ *n* = 6).

Histone acetylation at H3K9 was significantly increased in SIRT6^+/–^ mice compared to SIRT6^+/+^ control mice (Figures 2A–C). Increases in histone acetylation in SIRT6^+/–^ mice occurred in both endothelial and vascular smooth muscle cells of the vessel wall (see micrographs in Figure 2).

### Reactive Oxygen Species Levels (Lucigenin-Enhanced Chemiluminescence)

**FIGURE 3:**
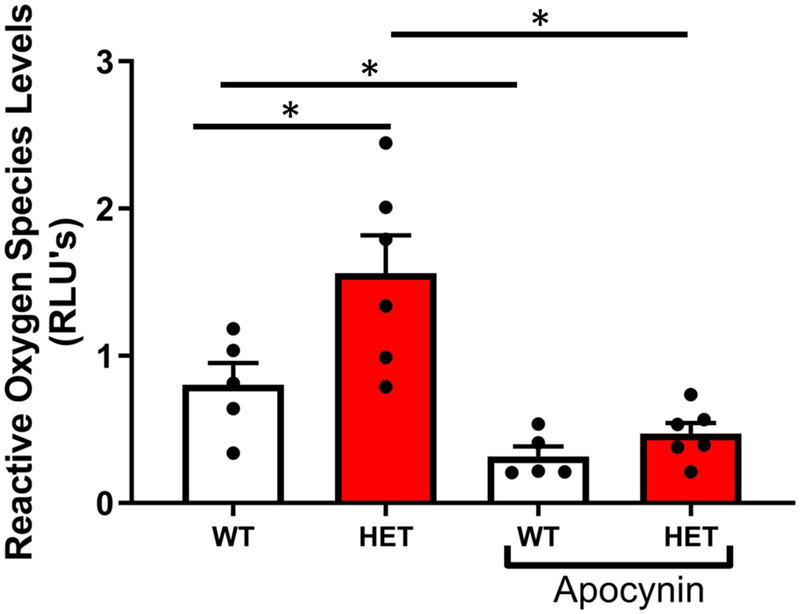
Changes in reactive oxygen species levels with SIRT6 haploinsufficiency. Note that superoxide levels are significantly increased in SIRT6^+/–^ mice compared to SIRT6^+/+^ mice (* denotes *p* < 0.05). Inhibition with apocynin, an inhibitor of NAD(P)H oxidase, significantly decreased reduced superoxide levels in both groups of mice (SIRT6^+/+^ *n* = 5 and SIRT6^+/–^ *n* = 6).

Superoxide levels were significantly increased in aorta from SIRT6^+/–^ compared to SIRT6^+/+^ littermates, and were significantly reduced by pre-incubation of vessel segments with apocynin (Figure 3).

### Vasomotor Function (Isolated Organ Chamber Bath)

**FIGURE 4:**
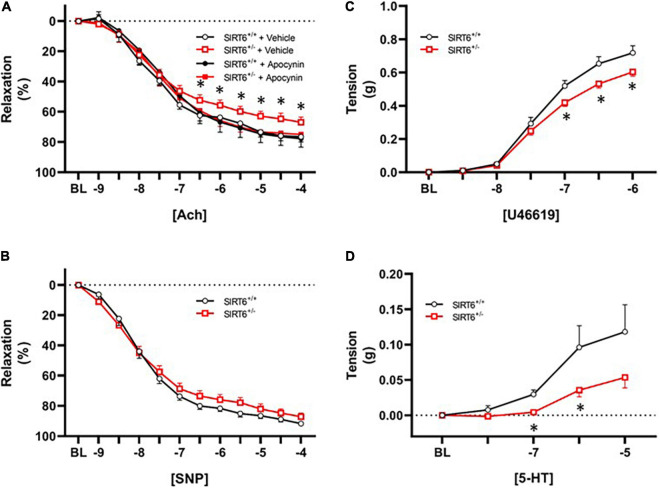
Changes in endothelial function in SIRT6 haploinsufficient mice. **(A)** Responses to acetylcholine in SIRT6^+/+^ and SIRT6^+/–^ mice. Note that responses to acetylcholine are significantly impaired in SIRT6^+/–^ mice, and that vasodilation to acetylcholine is normalized by incubation of vessels with the NAD(P)H oxidase inhibitor apocynin. **(B)** Responses to sodium nitroprusside in SIRT6^+/+^ and SIRT6^+/–^ mice. While there is a rightward shift of the curve in SIRT6^+/–^ mice, this trend did not reach statistical significance. **(C)** Constrictor responses to the thromboxane receptor agonist U46619 in SIRT6^+/+^ and SIRT6^+/–^ mice. Note that peak force generation is significantly reduced in SIRT6^+/–^ mice. **(D)** Responses to serotonin in SIRT6^+/+^ and SIRT6^+/–^ mice. Note that contractile responses to serotonin are significantly impaired in SIRT6^+/–^ mice compared to controls (SIRT6^+/+^ *n* = 9 and SIRT6^+/–^ *n* = 12). * denotes *p* < 0.05.

Vascular relaxation in response to acetylcholine was significantly impaired in SIRT6^+/–^ mice compared to SIRT6^+/+^ control mice (67 ± 3% versus 76 ± 3%, respectively; *p* < 0.05, Figure 4A). Vasomotor responses to exogenous acetylcholine were virtually identical between groups when a subset of vessel segments were incubated with the nitric oxide inhibitor L-NAME (36 ± 7% versus 36 ± 9%, respectively; p = n.s. with *n* = 4/group). Short-term incubation (30 min) of vascular rings with the NAD(P)H oxidase inhibitor apocynin significantly improved vascular relaxation to acetylcholine only in SIRT6^+/–^ mice (Figure 4A). Responses to sodium nitroprusside were similar between SIRT6^+/+^ and SIRT6^+/–^ mice (Figure 4B). Maximum tension in response to U46619 was significantly reduced in SIRT6^+/–^ mice compared to SIRT6^+/+^ control mice (Figure 4C), and contractile responses to serotonin were also significantly attenuated at multiple doses in SIRT6^+/–^ mice compared to controls (Figure 4D).

## Discussion

The key findings of this study are: (1) reduction of SIRT6 increases histone acetylation in conduit vessels, (2) reduction of SIRT6 increases production of NAD(P)H oxidase-derived ROS in conduit vessels, and (3) reduction of SIRT6 impairs endothelium-dependent relaxation in carotid arteries.

### Sirtuin 6 Regulates Histone Acetylation Levels in Aorta

In the present study, we used genetically altered mice to reduce SIRT6 expression by ∼50%, and found that inactivation of one copy of SIRT6 resulted in histone hyperacetylation at H3K9. While previous studies demonstrated that H3K9 and H3K56 are SIRT6 targets in several tissues and cell types *in vivo* and *in vitro* (Mostoslavsky et al., 2006; Kawahara et al., 2009; Michishita et al., 2009), to our knowledge, this is the first study to demonstrate that SIRT6 is expressed at physiologically relevant levels and targets these sites in vascular tissue.

Our goal was to reduce SIRT6 without affecting expression of other SIRT isoforms. The observation that SIRT1 expression was unchanged was particularly important given previous reports that SIRT1 is an important determinant of protein/histone acetylation (Donmez and Guarente, 2010), eNOS activity (Mattagajasingh et al., 2007), NFκB signaling (Yeung et al., 2004; Salminen et al., 2008), and gross vascular pathology in aorta (Donato et al., 2011). Despite overlap between SIRT1 and SIRT6 nuclear targets (e.g., H3K56), the data from the current study and others (Mostoslavsky et al., 2006; Salminen et al., 2008; Michishita et al., 2009; Yang et al., 2009) would suggest that there is not a functional or sufficient compensatory increase in overall sirtuin activity when levels of one isoform are reduced.

We did, however, observe reductions in SIRT2 and SIRT4 in aorta from SIRT6-deficient mice. There are three lines of evidence suggesting that these changes are unlikely to explain the functional and molecular changes we observed with reduction of SIRT6. First, changes in expression of SIRT2 and SIRT4, while statistically significant, are relatively small. Second, SIRT2 has been shown to play a deleterious role in the induction of many pathophysiological processes (Narayan et al., 2012). Thus, we would anticipate that reductions in SIRT2 would be protective, and would only serve to mask the true physiological impact of reducing SIRT6. Third, SIRT4 is localized predominantly in mitochondria (Sack and Finkel, 2012), and thus cannot explain the increases in nuclear histone acetylation we observed in the present study.

### Reduction of Sirtuin 6 Increases Reactive Oxygen Species and Impairs Endothelial Function

Our data demonstrate that reduction of SIRT6 significantly increases superoxide levels in aorta. While a net change in superoxide levels is a balance between production and dismutation of superoxide (Csiszar et al., 2007; Alom-Ruiz et al., 2008), endothelial dysfunction in contexts such as hypertension, atherosclerosis, and aging is often due to increases in NAD(P)H oxidase activity (Dworakowski et al., 2006, 2008b; Heistad et al., 2009). Mechanistically, increases in superoxide and/or hydrogen peroxide limit endothelium-dependent vasodilation through conversion of nitric oxide to peroxynitrite, which does not elicit vasodilation and can nitrosylate and inactivate other antioxidant proteins (Miller et al., 2008, 2010). Furthermore, increases in reactive oxygen species can oxidize soluble guanylate cyclase, a major downstream effector involved in NO-dependent vasodilation (Bachschmid et al., 2005; Schildknecht and Ullrich, 2009).

Our final key finding in the current report is that endothelial function in SIRT6^+/–^ mice was normalized by acute incubation of vessels with apocynin, which reduces NAD(P)H oxidase activity through inhibiting assembly of the NADPH oxidase complex (Stolk et al., 1994). Our data showing identical vasomotor responses to acetylcholine in vessels pre-treated with the nitric oxide synthase inhibitor L-NAME suggests that other endothelium-dependent vasodilatory mechanisms are largely unchanged by SIRT6 deficiency, and suggest the changes observed herein are largely due to reductions in nitric oxide bioavailability and signaling (Feletou and Vanhoutte, 2006). Previous studies suggested that SIRT1 tonically suppresses expression of NAD(P)H oxidase subunits, and while this has clear implications for influencing oxidative state and nitric oxide signaling, mechanisms whereby this occurs have not been clearly elucidated. One plausible link between SIRT1/6 activity and NAD(P)H oxidase expression is through modulation of NF-κB signaling (Yeung et al., 2004; Salminen et al., 2008; Li et al., 2009; Edderkaoui et al., 2011; Ungvari et al., 2011), which is tonically suppressed through the maintenance of low histone acetylation levels by SIRT1 and SIRT6 (Michishita et al., 2008; Salminen et al., 2008; Kawahara et al., 2009).

### Limitations

As this is a highly focused brief report, there are several limitations to this work. First, while we have identified activation of NADPH oxidase as a potential mechanism contributing to endothelial dysfunction in this model, we did not identify the specific mechanistic steps whereby SIRT6 induces this phenomenon. This is largely due to the potential for a vast number of molecular changes that can be induced with inactivation of one copy of SIRT6 and subsequent histone hyperactetylation and genomic instability. Furthermore, we only studied haploinsufficient mice herein, as knockout of SIRT6 in our experience results in dramatic blunting of animal growth and high mortality, and removal of aorta from such mice was not successful without significant damaging of the vessel. Nevertheless, prior work suggests that induction of premature senescence is a consequence of SIRT6 inactivation in vitro (Lee et al., 2020), which can subsequently increase Nox2 activity (Huang et al., 2017) (a phenomenon demonstrated in kidney). Ultimately, we feel these are exciting new directions for investigation in the field moving forward.

### Conclusion

In summary, this is the first study to demonstrate that global-genetic reductions of SIRT6 are a major determinant of histone acetylation and oxidative stress in the vasculature, and that deficiency of SIRT6 results in significant endothelial dysfunction due to increases in NAD(P)H oxidase-derived reactive oxygen species. Given the large amount of literature implicating endothelial dysfunction in disease contexts ranging from aging to atherosclerosis to hypertension, our data suggest that activators of SIRT6 could represent a novel class of therapeutic compounds with broad clinical utility.

## Data Availability Statement

The raw data supporting the conclusions of this article will be made available by the authors, without undue reservation.

## Ethics Statement

The animal study was reviewed and approved by Mayo Clinic Institutional Animal Care and Use Committee.

## Author Contributions

LG: data acquisition and analysis and manuscript drafting. BZ and CR: data acquisition and analysis, manuscript drafting, and revision. MH: data acquisition and analysis. F-PJ: data acquisition and data interpretation. JM: study design, data interpretation, manuscript drafting, and revision. All authors contributed to the article and approved the submitted version.

## Conflict of Interest

The authors declare that the research was conducted in the absence of any commercial or financial relationships that could be construed as a potential conflict of interest.

## Publisher’s Note

All claims expressed in this article are solely those of the authors and do not necessarily represent those of their affiliated organizations, or those of the publisher, the editors and the reviewers. Any product that may be evaluated in this article, or claim that may be made by its manufacturer, is not guaranteed or endorsed by the publisher.
